# Effects of Cadmium Exposure on the Immune System and Immunoregulation

**DOI:** 10.3389/fimmu.2021.695484

**Published:** 2021-07-20

**Authors:** Zhineng Wang, Ying Sun, Wenbo Yao, Qian Ba, Hui Wang

**Affiliations:** ^1^ School of Food and Biotechnological Engineering, Shaanxi University of Science and Technology, Xi’an, China; ^2^ State Key Laboratory of Oncogenes and Related Genes, Center for Single-Cell Omics, School of Public Health, Shanghai Jiao Tong University School of Medicine, Shanghai, China

**Keywords:** immunoregulation, adaptive immunity, innate immunity, cadmium, toxicity

## Abstract

Cadmium (Cd), a biologically non-essential heavy metal, is widespread in the environment, including the air, water, and soil, and is widely present in foods and quantum dot preparations. Cd enters the body primarily through inhalation and ingestion. Its biological half-life in humans is 10–35 years; therefore, Cd poses long-term health risks. While most studies on Cd toxicity have focused on organ and tissue damage, the immunotoxicity of Cd has drawn increasing attention recently. Cd accumulates in immune cells, modulates the function of the immune system, triggers immunological responses, and leads to diverse health problems. Cd acts as an immunotoxic agent by regulating the activity and apoptosis of immune cells, altering the secretion of immune cytokines, inducing reactive oxygen species (ROS) production and oxidative stress, changing the frequency of T lymphocyte subsets, and altering the production of selective antibodies in immune cells. This review summarizes the immunological toxicity of Cd, elucidates the mechanisms underlying Cd toxicity in terms of innate immunity and adaptive immunity, and discusses potential strategies to alleviate the adverse effects of Cd on the immune system.

## Introduction

Heavy metal elements occur naturally in the environment as organic or inorganic compounds. In addition, heavy metals are released into the ambient air or sewage network in various industrial and combustion processes and are subsequently deposited in soil and water, where they enter the food chain and can pose human health risks (1, 2). Over the past century, industrialization has progressed at a rapid pace, which has greatly increased the demand for the exploitation of Earth’s natural resources and led to global environmental pollution (3).

**Figure 1:**
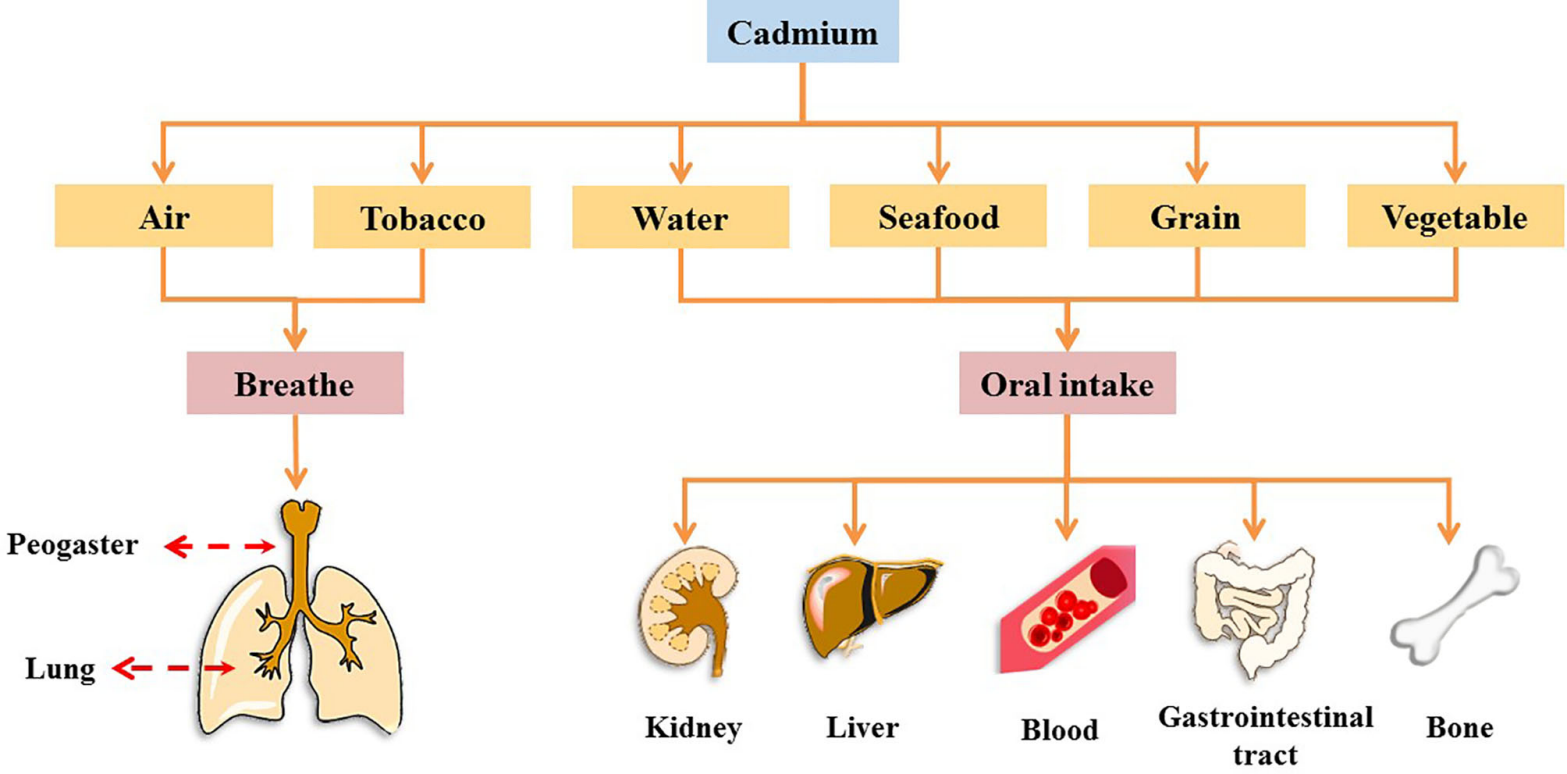
Major routes of Cd exposure and toxic effects of Cd on different organs in the human body.

Cadmium (Cd), a heavy metal, originates from both natural and anthropogenic sources. Geological weathering is the primary natural source of Cd, and anthropogenic sources include mining, smelting, wastewater irrigation, industrial and vehicular emissions, the deep burial of nickel-Cd batteries, manufacturing, and agrochemicals (4). In 1993, Cd was designated as a Class I carcinogen by the International Agency for Research on Cancer (IARC), and it is one of the worst heavy metal pollutants (5–7). It has no physiological function in the human body (8). Further, Cd cannot be metabolized by the human body and is difficult to excrete; as a result, it has a biological half-life of 10–35 years in humans (9). Humans are at high risk of Cd exposure through ingestion from water, grains, leafy vegetables, potatoes, and seafoods, as well as inhalation (7, 10). Occupational contact with Cd occurs primarily through respiration, whereas in the general population, Cd intake occurs primarily through food ingestion and recreational smoke inhalation (2, 11, 12). In many countries, Cd levels in humans have been found to exceed the tolerance of various organs and are associated with increased risk of chronic diseases, such as cancer, diabetes, and osteoporosis (13). Cd accumulation occurs in several organs and tissues. Cd causes acute or chronic toxicity in the lungs, kidneys, liver, and bones and can be absorbed into the blood from the lungs and gastrointestinal tract, where it binds to blood cells, thus affecting the blood system (**Figure 1** and **Box 1**) (5, 14–28).

Box 1 **Effects of cadmium exposure on peogaster, lung, kidney, liver, blood, gastrointestinal tract, and bone.**

1. Peogaster and lung
  Studies have shown that the acute toxic properties of Cd were observed through the air-liquid-interface airway tissue models, which led to induce aberrant mucins expression and secretion, impaired cilia function, and squamous differentiation, thus impairing the function of the mucosal barrier in the lung and peogaster (14).
2. Kidney
  Cd is a famous nephrotoxic substance. With Cd exposure, blood Cd in children, adolescents, and adults was positively associated with markers of kidney damage (as indicated by increased levels of N-acetyl-b-D-glucosaminidase and b2-microglobulin) (15). Therefore, the current exposure to low levels of Cd may have caused adverse reactions in renal tubules.
3. Liver
  After Cd induction, liver tissue was induced to have liver parenchyma with lymphocytes, fibrosis, microvesicular steatosis of the hepatocytes, hepatocellular micro-bubble fat degeneration, as well as many phagocytic cells, pyknotic cells, and vacuolation for Cd, thus producing toxic effects on the liver (16).
4. Blood
  Cd can interact with red blood cells, significantly reduce the activity of red blood cells superoxide dismutase and catalase, and increase the concentration of lipid peroxides (17).
5. Gastrointestinal tract
  The gastrointestinal tract is one of the targets of Cd action and causes an acute inflammatory response in the intestines of mice, causing villus damage to the intestinal tissue and accompanying the immersion of inflammatory cells (18). In addition, in the intestinal inflammation caused by Cd exposure, Cd intake was observed to alter the structure of the gut microbiome and reduce the relative abundance of lactobacillus in the intestine (19, 20).
6. Bone
  Cd exposure was associated with changes in bone metabolism and osteoporosis. There is growing evidence that Cd has a direct effect on osteoblasts, which accumulate and induce apoptosis in osteoblasts (21). As a result, Cd accumulation can have toxic effects on the bones, thereby reducing the density of bone minerals, regulating the expression of bone formation genes, affect the activity of osteoclasts and Ca absorption, thereby increasing the risk of osteoporosis (22, 23).

Cd mainly induces damage through the production of free radicals, which affect the mitochondrial activity and can induce apoptosis (29). Cao et al. showed that Cd activates the mitochondria-mediated internal apoptosis pathway in BEAS-2B cells, thus reducing their viability, causing reactive oxygen species (ROS) accumulation, inducing apoptosis, suppressing B-cell lymphoma-2 expression, and enhancing B-cell lymphoma-2-associated X and cleaved caspase-3 protein expression (12). Pathak et al. showed that Cd-treated splenocytes and thymocytes produce large amounts of ROS, which not only serve as a key mediator of Cd-induced apoptosis but also damage the mitochondrial membrane.

Heat shock proteins are general markers of cell stress. In nucleated blood cells, especially lymphocytes, Cd enhanced the transcription of metallothionein-IIA and heat shock protein 70 genes in time- and dose-dependent manners upon treatment with CdCl_2_ at concentrations from 5–50 μmol/L (1, 30). In addition, Cd can directly damage DNA and disrupt the DNA repair process (16). As an efficient inducer of immune poisons and heat shock proteins, Cd triggers stress responses and exerts deleterious effects in immune cells (31).

(1) Cd accumulates in immune cells and dose-dependently induces apoptosis.
  Chatterjee et al. showed that Cd treatment in Swiss albino mice (2.5–40 μmol/kg body weight) led to dose-dependent Cd accumulation in splenocytes and induced apoptosis of splenic lymphocytes (32). Tsangaris et al. reported that Cd exposure induced apoptosis in the immune cell lines Raji, CCRF-CEM, and Molt-3 (30). Cd enters cells through the L-type calcium (Ca) channel, increases intracellular Ca concentrations, and binds to cytoplasmic and nuclear components, thus accumulates within cells (1, 33).
(2) Cd induces the differentiation of immune cells and changes the composition and proportion of lymphocyte subsets.
  Desforges et al. found that Cd exposure affects the development of immune organs, differentiation of immune cells, and specific and non-specific immune responses in marine mammals (34). In mice, Cd exposure affects the composition and proportion of lymphocytes, including CD4^+^, CD8^+^, CD25^+^, CD44^+^, and CD223^+^ cells (35–37). In addition, Cd treatment reportedly promotes DNA synthesis, the adherence capacity of macrophages and lymphocytes, and cell proliferation. For example, human fibroblasts and prostatic epithelial cells exposed to low doses of Cd can develop into malignant cells (38, 39). This further suggests that Cd can interfere with normal immune system growth and development.
(3) Cd induces inflammatory responses in immune cells by activating multiple signaling pathways.

Micromolar concentrations of Cd activate multiple signaling pathways, in particular, the nuclear factor kappa B (NF-κB) and mitogen-activated protein kinase (MAPK) pathways, in immune cells and induce the upregulation of inflammatory markers and mediators (36, 38, 40). In addition, Cd can induce ROS production, cause mitochondrial injury, reduce antioxidant enzyme activity, induce the oxidative stress response, and activate endoplasmic reticulum stress (ERS) pathways, thereby playing a proinflammatory role in immune cells (41–48). Thus, Cd affects various cell functions, including the regulation of immune-cell activity and the secretion of cytokines in innate and adaptive immunity.

In recent years, Cd toxicity research has mostly focused on the toxic effects of Cd on different organs, while the effects of Cd exposure on immune function and its role in immune-system regulation should be comprehensively investigated. We provide an overview of the mechanisms of innate and adaptive Cd immunotoxicity to provide new insights into the effect of Cd exposure on immunity, as well as effective ways to alleviate Cd cytotoxicity.

## Cd and the Immune System

The immune system is a highly evolved biological system that is susceptible to environmental influences. While most new substances and microorganisms are often harmless, a small fraction of cases can pose a high risk (49). An effective immune system must be able to discriminate these instances by distinguishing self from non-self and harmless non-self from dangerous non-self (50). Therefore, the immune system is responsible for immunologic surveillance and defense, and immunoregulation. The immune system is a complex network of immune organs (bone marrow, spleen, lymph nodes, tonsils, and thymus, etc), immune cells (lymphocytes, macrophages, neutrophils, eosinophils, and basophilic cells, etc), and immune-active substances (antibodies, lysozymes, complement factors, immunoglobulins, interferons, and other cytokines, etc), in which immune cells communicate with each other through direct interaction or soluble cytokines (51).

The immune system is divided into innate immunity and adaptive immunity (52, 53). Innate immunity refers to the normal physiological defense function of the body. As the first line of host defense, it is fast and non-specific. Innate immune cells are activated by danger signals, including pathogens and risk-related molecular patterns as well as metabolite-related warning signs. Innate immune activation can promote tissue inflammation or immune resolution directly through phagocytosis and the secretion of biologically active molecules or indirectly through activating the adaptive immune response mediated by antigen-presenting cells (54). Innate immunity is activated in response to invasion by different pathogenic microorganisms and foreign bodies, thus protecting the host from infectious microorganisms (55). The adaptive immune response is antigen-specific and forms the second line of host defense. Adaptive immunity includes cell-mediated [T lymphocytes (T-cells)] and somolytic [B lymphocytes (B-cells)] immunity, which plays vital roles in driving tissue inflammation and repair (54). The B-cell response is characterized by the production of antibodies, which play important roles in both innate and adaptive immunity. There are two types of B-cell immunity: T-cell-dependent and independent (56). The innate and adaptive immune systems work in concert to achieve the removal of senescent cells and defense against invading pathogens (57).

**Figure 2:**
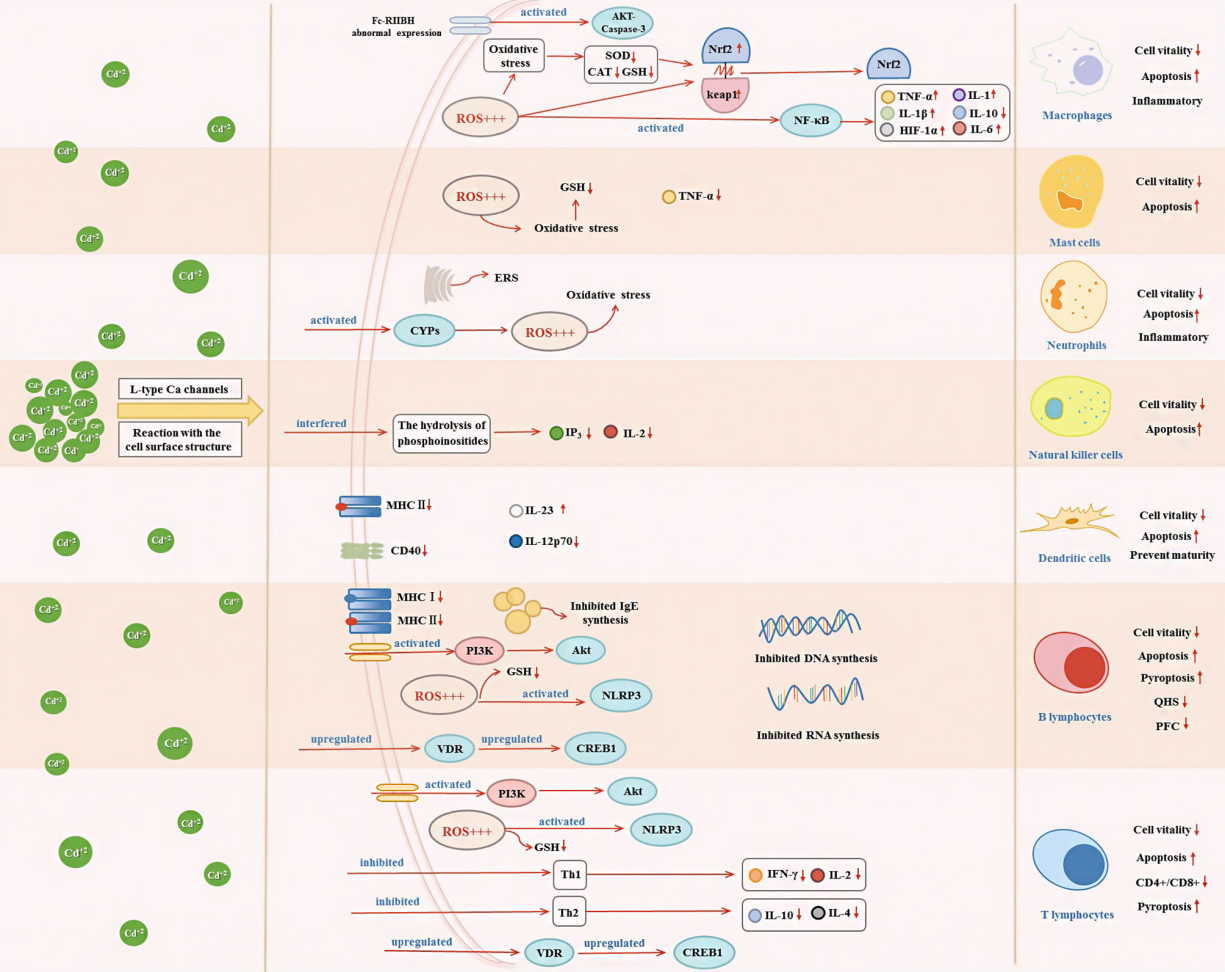
Effects of Cd exposure on innate immune and adaptive immune cells and their underlying mechanisms. Cd ions can enter cells through Ca channels of the L-type or react with surface structures of the cell. After entering the cell, Cd will affect the secretion of immune cytokines through activating oxidative stress, ERS, NF-κB, VDR/CREB, NLRP3, CYPs, and PI3K/Akt signaling pathway, which in turn decrease cell vitality and induce apoptosis.

Cd, as an immunotoxic inhibitor, interacts directly with immune cells and changes their status and functionality, thus damaging the immune system, in time- and dose-dependent manners (58). In what follows, we summarize the effects of Cd on innate and adaptive immune cells based on *in vitro* and *in vivo* experiments and the modes of action of Cd at different doses and exposure times (**Tables 1**, **2** and **Figure 2**).

**Table 1:** Effects of cadmium exposure on immune cells under experimental conditions *in vitro*.

Immune system	Immune cell	Cell line	Cd dose	Exposure duration	Cellular effects	Reference
Innate immunity	Macrophages	Mice peritoneal macrophages	8.0×10^-3^ mg/L; 8.0×10^-2^ mg/L; 8.0×10^-1^ mg/L	30 min	Phagocytic capacity was significantly reduced	(59)
		Rat lung macrophage line NR8383 cells	10 μg/mL	2 h; 24 h	The Fc-RIIB receptor level is abnormal and causes cell damage	(60)
		Chicken peritoneal macrophages	20 μM; 50 μM	12 h	Led to dose-dependent cytotoxicity and abnormal immune response	(46)
		Mouse alveolar macrophage cell lines	50 μM	3 h	Promoted increased macrophage glycolytic function with enhanced extracellular acidification rate, glycolytic metabolites, and lactate excretion	(61)
		Human acute monocytic leuke- mic THP-1 cell line	0.1 μM; 1.0 μM; 2.5 μM; 5.0 μM; 10 μM; 20 μM; 40 μM; 100 μM	4 h	Cd results in immune dysfunction in macrophages through inhibition of the NF-κB signaling pathway	(62)
		Mouse resident peritoneal macrophages	5 μM; 10 μM; 20 μM; 30 μM; 40 μM; 50 μM	18 h	Cd produce an important impact on arachidonic acid turnover in macrophages	(63)
		Murine macrophage-like cell line RAW 264.7	0.1 mM; 0.3 mM; 1 mM;3 mM	24 h	Cd exposure generated oxidative stress and decreased the inflammatory responses	(42)
		Murine Macrophage Cell Line RAW 264.7	0.01 μM; 0.1 μM; 10 μM	2 h	Cd alone caused a dose-dependent decreased viability of exposed cells	(64)
	Mast cells	Mouse mast cell MC/9	0.01 μM; 0.1 μM; 1 μM; 10 μM; 100 μM	24 h	Mast cells had full dose-response depletion of glutathione below cytotoxic levels and mast cells would be more susceptible to oxidative stress	(65)
	Neutrophils	Common carp neutrophils	10 µM	2 h	Cd-induced neutrophil apoptosis and immunosuppression	(44)
		chicken neutrophils	10^-6^ M	12 h; 24 h; 36 h; 48 h	Cd-induced immune suppression, inflammatory response, and apoptosis *via* endoplasmic reticulum stress	(41)
	Natural killer (NK) cells	K562 cells	100 μM	1 h	Cd inhibited the cytotoxic activities of effector cells prestimulated with IL-2, which mostly consist of NK cells	(66)
Adaptive immunity	T-lymphocyte (T-cells)	Male BALB/c mice thymocyte	10 mM; 25 mM; 50 mM	6 h; 12 h; 18 h	Cd-induced T-cell apoptosis and changes the CD4^+^/CD8^+^ ratio	(36)
		Mice spleen cells	10 μM	24 h	Cd exposure suppresses the proliferation of T-cells	(67)
		T-cells were isolated from healthy human	0.003 µM; 0.03 µM; 0.33 µM; 3.33 µM; 33.33 µM; 66.66 µM	20 min; 40 min; 60 min; 90 min; 120 min	cadmium depleted T lymphocytes GSH to a harmful extent	(68)
		Peripheral blood mononuclear cells were isolated from healthy donors	0.01334 μM; 0.04448 μM; 0.1334 μM; 0.4448 μM 1.334 μM; 4.448 μM 13.34 μM 44.48 μM 133.4 μM 444.8 μM	24 h	Cd polarizes the immune response toward type-2 in cells stimulated *via* T-cell receptors	(33)
	B-lymphocyte (B-cells)	Female DBA/2J mice B-cells	0.1 μM; 1 μM; 10 μM; 100 Mm;10000 μM	3 h	Cd has an early inhibitory effect on B-cell activation	(69)
		Peripheral blood mononuclear cells were isolated from healthy adult	5 μM; 25 μM; 50 μM	24 h; 48 h	Only IgE but not IgG synthesis of purified B-cells were inhibited by Cd	(1)
		Peripheral blood mononuclear cells were isolated from healthy adult	0.01 μM; 0.1 μM 1 μM; 2 μM 5 μM; 10 μM	24 h; 72 h; 144 h; 14 d	The viability of B-cells decreases with the increase of Cd concentration	(70)
		B-cell line Raji	5 μM; 10 μM 15 μM; 20 μM 25 μM; 30 μM 35 μM; 40 μM 50 μM; 75 μM 100 μM	18 h	Cd-induced apoptosis in a dose-dependent manner in the Raji B-cell line	(30)
		Human Ramos B cells	0. 1 μM; 1 μM 2.5 μM; 5 μM	24 h	Cd exposure induced apoptosis, which was dependent on Cd-induced vacuole membrane protein 1 expression and autophagy	(71)
		B-cells were isolated from healthy human volunteer blood sample	0.003 µM; 0.03 µM; 0.33 µM; 3.33 µM; 33.33 µM; 66.66 µM	20 min; 40 min; 60 min; 90 min; 120 min	cadmium depleted B lymphocytes GSH to a harmful extent	(68)

**Table 2:** Effects of cadmium exposure on immune cells under experimental conditions *in vivo*.

Immune system	Immune cell	Animal	Cd dose	Exposure duration	Cellular effects	Reference
Innate immunity	Macrophages	Male Japanese quail	50 ppm; 100 ppm; 150 ppm	4 weeks	Cytokine expression (IL-1β, IL-6, and TNF-α) and phagocytosis activity was reduced	(72)
		WT C57BL6 mice	100 ng/kg	7 d	Cd mediates the persistence of classically activated lung macrophages to exacerbate lung injury	(61)
		*Channa punctatus* Bloch	1.96 mg/L	7 d	Cd-induced oxystress triggers apoptosis *via* both mitochondrial and death receptor pathways	(73)
		*Channa punctatus* Bloch	1.96 mg/L	7 d	The decreased phagocytosis, intracellular killing, and cell adhesion were significantly reduced	(74)
		Male Balb/c mice	15 ppm	2 months	Cd exposure altered the redox balance, leading to excessive production of reactive oxygen species that overwhelmed the antioxidant defenses	(43)
	Neutrophils	Male Dark Agouti rats	5 ppm;50 ppm	1 month	Cd promotes neutrophil proliferation	(75)
	Natural killer (NK) cells	Male Dark Agouti rats	5 ppm;50 ppm	1 month	The number of NK cells in the spleens of rats decreased	(75)
		male C57BL/6 mice	50 ppm	3 weeks	Cd-treated mice had significantly lower Nk cell activity	(76)
		Wistar female rats	200 ppm; 400 ppm	170 d	Cd induces both inhibitory and stimulatory effects on rat NK cell number and cytotoxic activity	(77)
	Dendritic cells (DCs)	Male Swiss Albino mice	2.5 mg/kg; 5 mg/kg; 7.5 mg/kg; 10 mg/kg; 15 mg/kg	4 weeks	Cd affects DCs maturation and function	(6)
Adaptive immunity	T-lymphocyte (T-cells)	C57Bl/6 mice	10 ppm	7 weeks	CD8^+^CD223^+^ T-cells were markedly decreased	(35)
		Male Sprague Dawley rats	35 ppm	10 weeks	Cadmium exposure also significantly increased the production of IFN-γ, and IL-10, and may affect multiple T cell subsets.	(78)
		Male Sprague- Dawley rats	5 ppm; 10 ppm; 25 ppm; 50 ppm; 100 ppm	1 month	Cd can cause changes in CD4^+^ and CD8^+^ cells numbers	(25)
		Male Dark Agouti rats	1 mg Cd/kg	30 d	Differential effects on proinflammatory T-cell derived cytokines were observed (decreases of IFN-γ gene expression and ConA-stimulated production	(75)
	B-lymphocyte (B-cells)	Male Sprague- Dawley rats	5 ppm; 10 ppm; 25 ppm; 50 ppm; 100 ppm	1 month	Low concentrations of Cd (5 ppm and 10 ppm) reduced the number of B-cells, while high concentrations of Cd (25 ppm, 50 ppm, and 100 ppm) increased the number of B-cells	(25)
		Male ICR mice	0.5 mg Cd/kg; 1 mg Cd/kg	5 d	The decrease of blood B lymphocytes is accompanied by the increase in the number of splenic B lymphocytes	(79)
		Female mice	5 µg/mL; 10 µg/mL; 50 µg/mL	4 weeks	Dose-dependent enhancement of B lymphocyte activity and Cd concentration	(28)

## Regulatory Effects of Cd on Innate Immunity

Long-term exposure to low doses of Cd results in Cd accumulation in innate immune cells. Once Cd has entered the cell, it tends to occupy metal-binding protein domains, replacing essential metals (co-factors) in enzymes and thus inhibiting their ability to maintain cell function (2). However, apoptosis (programmed cell death) is an important feature of Cd toxicity. Many innate immune cells and mechanisms facilitate self- and non-self-recognition (80). Innate immune cells include monocytes, macrophages, neutrophils, natural killer (NK) cells, and dendritic cells (DCs) (2). Cd exposure affects multiple aspects of innate immunity by regulating innate immune responses, including chemokine expression and release (5, 81).

### Cd and Macrophages

Macrophages function in identifying and eliminating non-self entities and thus play important roles in innate immunity and inflammation processes (60). Phagocytosis and endocytosis are regulated through specific receptors on the macrophage surface. In particular, the expression of Fc-gamma receptor II (Fc-RIIB) on the surface of macrophages is related to their immunoprotective function. Wu et al. reported that macrophages exposed to Cd *in vitro* have abnormal surface levels of Fc-RIIB, resulting in severe cell damage (60). In addition, Loose et al. showed that the phagocytic capacity of murine peritoneal and pulmonary alveolar macrophages was significantly reduced by Cd at concentrations of 8.0 × 10^–3^, 8.0 × 10^–2^, and 8.0 × 10^–1^ mg/L (59). Macrophages are classified as classically activated (M1) or alternatively activated (M2) based on their pro- or anti-inflammatory phenotype, respectively. The proportion of these two subtypes plays a crucial role in tissue inflammation, injury, and repair (82).

#### Cd and M1 Macrophages

In response to lipopolysaccharide (LPS) and Toll-like receptor signaling, M1 macrophages polarize and secrete a range of pro-inflammatory cytokines, such as interleukin (IL)-1β, IL-23, IL-12, and tumor necrosis factor (TNF)-α (83). A recent study showed that LPS activates immunity-related processes in macrophages, whereas Cd inhibits these processes (62). An *in-vivo* study by Zhang et al. showed that Cd (20 μM and 50 μM) dose-dependently caused morphological and ultrastructural damage in chicken peritoneal macrophages, ROS accumulation, and mitochondrial injury (46). Furthermore, Cd exposure inhibited the activity of chicken peritoneal macrophages and promoted the expression of IL-1β, IL-6, and TNF-α in both inactivated macrophages and cells in response to LPS stimuli (46). Similarly, in mammals, long-term Cd exposure can result in reduced expression of cytokines, such as IL-1 and TNF-α, and inhibition of macrophage activity (72). These cytokines are produced by macrophages and can be used as markers for macrophage activity (42, 84). In addition, MAPK pathway activation and NF-κB-dependent gene expression can be used as indicators of macrophage proliferation, as demonstrated in an *in-vitro* study (5). In a study by Misra et al., Cd (1 μM) induced DNA synthesis in macrophages, activated the MAPK and NF-κB signaling pathways, and promoted macrophage proliferation, thereby inducing a malignant phenotype (38). In fact, under complex pathological conditions, macrophages can exhibit mixed phenotypes and polarize to a predominant phenotype depending on the duration and stage of injury and/or repair. Larson-Casey et al. showed that following Cd (100 ng/kg)-induced lung injury in mice, lung macrophages polarized to a pro-inflammatory M1 phenotype (61). Cd-mediated mitochondrial ROS generation induced NF-κB activation, increased the expression of hypoxia-inducible factor 1α (HIF-1α), and mediated the persistence of M1 lung macrophages to exacerbate lung injury (61). Choudhury et al. showed that in macrophages of *Channa punctatus* Bloch, Cd (1.96 mg/L) exposure induced inflammation by inhibiting the NF-κB pathway and altering nuclear factor erythroid 2-related factor 2 (Nrf2)-Kelch-like ECH-associated protein 1 (Keap1) signaling (73, 74). *In-vitro* experiments conducted by Cox et al. revealed that Cd (0.1–100 μM) induced immune dysfunction in macrophages by inhibiting NF-κB signaling (62). Cd also induces macrophages to promote inflammation through activating protein kinase B (Akt)-caspase-3 signaling (60) (**Figure 2**).

Cd accumulates and induces toxic effects in macrophages (85). Mouse osteoblasts exposed to Cd (1 mM) showed increased gene expression of macrophage migration inhibitory factor to prevent upregulation of macrophage growth factor expression, increasing the toxic effect of Cd on osteoblasts (21). Inflammatory diseases induce oxidative stress through enhanced free radical production in the body, which often results in increased oxidative damage. In immune organs, oxidative stress inhibits immune function (47, 86). Cd has been shown to induce oxidative stress and trigger adaptive cellular responses in mammalian cells (63). Therefore, oxidative stress is a potential mechanism by which heavy metals cause immune system disorders. Cd-induced oxidative stress may suppress the inflammatory response of macrophages; Cd exposure (0.3, 1, and 3 mM) induced oxidative stress and reduced the inflammatory response of mouse macrophages *in vitro* (42). This suggests that Cd exposure can inhibit the immune and oxidation systems. Similar findings *in vivo* have been reported by Ramirez et al.; in mice, long-term exposure to Cd (15 ppm) altered the reduction-oxidation balance in mouse peritoneal macrophages, leading to excessive ROS production (43). Numerous studies have demonstrated that oxidative stress plays an important role in the physiological regulation of macrophages in Cd-exposed mice (61, 72, 74).

#### Cd and M2 Macrophages

M2 macrophages are classified into three subpopulations, M2a, M2b, and M2c, all of which are primarily involved in tissue repair and respond to different stimuli (83). The secretion of anti-inflammatory factors, such as IL-4 and IL-10, is considered a hallmark of M2 polarization (87). M2 macrophages play an important role in the regulation of inflammation at infection and tissue damage sites by inhibiting inflammatory responses and regulating tissue repair processes and angiogenesis. Riemschneider et al. found that IL-10 expression was inhibited in macrophages from mice exposed to subtoxic doses of Cd (10 μM) (64).

Fatty acids play various roles in immune cells. Macrophage behavior and the inflammatory state are influenced by fluctuations of fatty acid levels (88). Arachidonic acid is a long-chain saturated fatty acid with anti-inflammatory activity found in phospholipids of mouse peritoneal macrophages. Free arachidonic acid is involved in degranulation, phagocytosis, adhesion, and cell proliferation. In mice, Cd (10 mM) exposure promoted the release of ROS in macrophages and the uptake of arachidonic acid, which may inhibit the M2 polarization of macrophages and reduce the anti-inflammatory activity of macrophages (63, 89).

### Cd and Mast Cells

In mammals, the innate immune system is responsible for controlling and limiting the progression of early infections. Mast cells are distributed throughout the body, generally participate in humoral and antibody-mediated immune responses, and play an important role in the first line of immunity (90). Cd toxicity modes, such as oxidative stress, depletion of antioxidants, and induction of apoptosis, have different outcomes, depending on the sensitivity of each cell type to different mechanisms (65). García-Mendoza et al. reported that under Cd exposure *in vitro*, mouse mast cells showed full dose-response depletion of glutathione (GSH) to below cytotoxic levels. In contrast, LPS-induced TNF-α and immunoglobulin (Ig)E-mediated histamine release in mast cells were attenuated by Cd (65) (**Figure 2**). Therefore, under Cd exposure, mast cells are more sensitive to oxidative stress.

### Cd and Neutrophils

Another major component of innate immunity is barrier defense, which involves epithelial and endothelial cells. In general, the innate immune system does not discriminate between harmful memory signals. Therefore, inflammation in Cd-containing cells reoccurs repeatedly, changing the recurrence cycle and exacerbating Cd toxicity over time. This occurs through the activation or inhibition of signaling pathways that alter the expression of anti- and pro-inflammatory mediators (2).

Neutrophils are important participants in the early response to pathogens and acute inflammation. Given the involvement in response to various invading pathogens and the regulation of innate and adaptive immune processes, the malfunction of neutrophils may play an important role in the pathogenesis of many diseases (91). Acute Cd (5 ppm and 50 ppm) treatment exerted an inflammatory effect in rats and increased the number of neutrophils in their spleens (75). Jiaxin et al. reported that Cd (10 µM) triggered the cytochrome P450s (CYPs) pathway and impaired antioxidant activity, leading to apoptosis and immunosuppression of neutrophils, in the common carp (44) (**Figure 2**). Cd-induced neutrophil apoptosis occurs *via* not only the mitochondria-dependent pathway but also ERS pathway (92). An *in-vivo* study by Chen et al. showed that Cd (1 μM) induced immunosuppressive and inflammatory reactions in chicken neutrophils and triggered apoptosis *via* the ERS pathway (41) (**Figure 2**).

### Cd and NK Cells

NK cells are lymphocytes of the innate immune system that play key roles in early antiviral responses, the secretion of cytokines, and the destruction of virus-infected cells (93). Cd targets NK cells in rats. Demenesku et al. found that the number of NK cells in the spleens of rats decreased after acute Cd (5 ppm and 50 ppm) treatment. And Chowdhury et al. showed that Cd (50 ppm) exposure reduced the activity of NK cells (75, 76). In rats, Cd (100 μM; 200 ppm and 400 ppm) ingestion suppressed the number of NK cells and exerted time-dependent toxic effects on NK cells (66, 77). In addition, phosphoinositide turnover as a signaling pathway in the activation of NK cells by NK-sensitive tumor target cells. Cd exposure can interfere with the hydrolysis of phosphoinositides, such as the decrease of inositol 1,4,5-trisphosphate (IP_3_) (66). Cd exposure also reduced IL-2 cytokine expression (66) (**Figure 2**). These reports indicate that Cd has a toxic effect on NK cells.

### Cd and DCs

DCs are antigen-presenting cells that bridge the innate and adaptive immune systems (6). They have efficient cellular uptake processes because immature DCs are located in peripheral tissues and continuously monitor the environment through the uptake of particulate and soluble products. Antigen-loaded DCs acquire a mature phenotype, which is associated with reduced endocytic and phagocytic capacities and enhanced production of inflammatory cytokines and chemokines. Mature DCs migrate to the lymphatic organs where they interact with and activate naïve T-cells (94, 95). Thus, DCs play a pivotal role in immune homeostasis and act as the primary regulator of immune system processes, including the induction of tolerance and prevention of autoimmunity (96, 97).

The ingestion of functional Cd-containing quantum dots results in minor cytotoxicity and inhibition of DC maturation. Chakraborty et al. reported that chronic CdCl_2_ (2.5–15 mg/kg body weight) exposure reduced the expression of key surface molecules, such as major histocompatibility complex (MHC) class II molecules and CD40, in bone-marrow-derived DCs of mice (6, 95) (**Figure 2**). CD40 induces DC maturation, and the decrease in CD40 expression indicated impaired DC maturation. In addition, the release of IL-12 by LPS-activated (IL-12p70) from bone-marrow-derived DCs was reduced, while IL-23 was increased upon CdCl_2_ exposure (6) (**Figure 2**). Therefore, Cd acts as an immunosuppressant by hindering DC maturation.

## Regulatory Effects of Cd on Adaptive Immunity

### Cd and T-Cells

T-cells are basic immune system cells that play a vital role in the cell-mediated adaptive immune response (90). The thymus is the site of T-cell differentiation and maturation. Considering that Cd causes DNA damage and that DNA repair by non-homologous end-joining is required for T-lymphocyte differentiation, it could be expected that Cd affects T-lymphocyte differentiation through this mechanism. However, Viau et al. showed that Cd does not affect non-homologous end-joining or base and nucleotide repair, but that Cd toxicity in T-cells is linked to cell-cycle perturbations (98). Cd intake by thymocytes altered the expression of thymocyte surface markers in mice, leading to phenotypic changes, and the lowest Cd concentration used in the study (10 mM) induced changes in different T-cell subsets. In mice, Cd treatment resulted in dose- and time-dependent accumulations of Cd in CD4^+^ cells and dose- and time-dependent decreases in the CD4^+^/CD8^+^ ratio, which is a bio-indicator of immunotoxicity (36). This immunosuppression is likely to result from reduced expression of IL-2 and interferon (IFN)-γ in T-helper (Th)1 cells and reduced expression of IL-4 in Th2 cells (36). In addition, Cd treatment causes abnormal immune phenotypes in the mouse uterus. Prenatal Cd exposure affected the transcriptomes of T-cells and CD4^+^ cells and disrupted cAMP-responsive element-binding protein 1 (CREB1) signaling, which is involved in T-cell stability (99) (**Figure 2**). When Cd stimulates T-cell subsets, T lymphocytes secrete cytokines, which further regulate the cellular immune response. Additionally, IFN-γ, TNF-α, and IL-2 secreted by Th1 lymphocytes inhibit the proliferation of Th2 lymphocytes, while IL-4, IL-5, IL-6, and IL-10 are secreted by Th2 lymphocytes inhibit the proliferation of Th1 lymphocytes. Cd exposure decreased cytokine production in Th1 (e.g., IFN-γ and IL-2) and Th2 (e.g., IL-4) lymphocytes (35, 100, 101) (**Figure 2**). Cd (35 ppm) affected multiple T-lymphocyte subsets and promoted the expression of inflammatory factors, thereby enhancing the inflammatory response (78). Lafuente et al. analyzed the dose-dependent effects of Cd on blood lymphocyte subsets and found that CD4^+^ and CD8^+^ cell counts decreased at doses of 5 and 10 ppm but increased at a dose of 25 ppm (25) (**Figure 2**).

Cd exposure at a dose of 1 mg/kg stimulated innate immune responses in rats; however, respiratory-burst stimuli inhibited T-cell responses (75). In addition, for proliferation, T-cells require zinc (Zn), which has chemical properties similar to those of Cd. Therefore, under Cd exposure, Zn intake by T-cells can be inhibited, resulting in the suppression of T-cell proliferation (67). This phenomenon had been reported before (102, 103). The regulatory effects of Cd on humoral immunity depend on the activation of cells. Cd polarizes the immune response toward type 2 in cells stimulated *via* T-cell receptors. Exposure of activated T-cells to low doses of Cd led to suppression of early Th1 cytokine events and suppression of the Th2 cytokines IL-4 and IL-10 (33) (**Figure 2**).

### Cd and B-Cells

Heavy metals can affect cells in two ways: by penetrating the interior of the cell through L-type Ca channels and by reacting with the surface structures of the cell (1). Cd affects B lymphocytes through both these ways (1). B-cell activity results in changes in protein expression on the surfaces of specific cells. In female mice, CdC1_2_ exposure affects the expression of surface antigens in B cells; for example, it inhibits the expression of class I MHC antigen and class II MHC antigens and inhibits the synthesis of B-cell RNA and DNA (69) (**Figure 2**).

B lymphocytes differentiate and mature in the spleen. Humoral immunity is an immune mechanism through which B lymphocytes are stimulated to produce antibodies that act as effectors of the humoral immune response for protection (104). When humoral immunity is inhibited, antibody-producing B lymphocytes are also inhibited. Cd induction significantly reduced humoral immunity parameters as well as plaque-forming cells and quantitative hemolysis of sheep red blood cell responses, resulting in humoral immunity suppression (104). In addition, Cd alters the signaling of B lymphocytes by stimulating the secretion of specific antibodies. When cells are stimulated to produce specific reactions, they respond to the synthesis of different proteins (1). Marth et al. used blood samples from nine healthy adult human donors to show that Cd (5, 25, and 50 µM) induced the transformation of the IgE antibody, produced by B lymphocytes, into IgG (1). In healthy nonallergic volunteers, Cd (0.1, 1, 2, 5, and 10 µM) significantly inhibited B-lymphocyte activity in a concentration-dependent manner and selectively inhibited IgE synthesis (70). These data show that the mechanism of action of Cd in activated B lymphocytes involves pathways that interrupt the effective initiation of cell activation and transduce a cytotoxic signal.

Numerous *in-vitro* studies have shown that Cd can induce apoptosis in various cell types (29). For example, CdCl_2_ at doses of 5 and 10 ppm induced apoptosis in peripheral blood B lymphocytes, and Cd at 5–100 µM induced apoptosis in Raji B cells in a dose-dependent manner (25, 30). Subcutaneous injections of Cd (0.5 and 1.0 mg/kg) in mice led to a significant reduction in blood B lymphocyte numbers (79). Cd is immunotoxic to B lymphocytes, and Cd (0.1–5 μM) exposure induced autophagy in B lymphocytes, thereby promoting apoptosis of immune cells (71). Cd (40 μM) exposure promoted apoptosis by activating inflammasome NOD-like receptor protein 3 (NLRP3) and promoted pyroptosis in splenic lymphocytes of carp fish (105). Cd caused apoptosis of porcine splenocytes through oxidative stress and activated the vitamin D receptor (VDR)/CREB1 pathway (106). Thus, these immunological alterations, activity changes, and apoptosis induction in T and B lymphocytes may be related to oxidative stress levels (e.g., they are correlated with GSH content). Ullah et al. reported that Cd (0.003–66.66 µM) exposure destroyed the antioxidant defense system and depleted GSH in T and B lymphocytes to a harmful extent (68). Cd (3.5 × 10^−5^ M) exposure promoted an imbalance of the antioxidant status and activated the phosphoinositide 3-kinase (PI3K)/Akt pathway, along with a decrease in GSH levels, eventually leading to T- and B-lymphocyte apoptosis (99, 107) (**Figure 2**).

## Potential Ways to Alleviate Cd Cytotoxicity

Cd toxicity is not limited to occupationally exposed workers but also causes moderate to severe health problems in individuals exposed to non-occupational sources of Cd. When Cd enters the human body, it is rapidly transported to various organs *via* blood circulation. Furthermore, the half-life of Cd in the human body is estimated to be in decades. Cd exposure at concentrations in the micromolar range can lead to significant toxic effects (6). Immune cells develop from hematopoietic stem cells in the bone marrow. In mice, Cd (10 ppm) interacted directly with hematopoietic stem cells, impairing their function *via* the activation of noncanonical Wnt signaling, which in turn affects immune function (108). Cd (30, 100, and 300 ppm) exerted significant immunosuppressive effects on humoral and cell-mediated immune responses in mice (109). Further, acute cadmium exposure can induce inflammatory diseases. For example, in rheumatoid arthritis, acute Cd exposure leads to a significant reduction in the T-to-B-cell ratio, which may produce immune-sensitizing effects (110).

(1) Prevent or mitigate the outcomes of Cd immunotoxicity

The scientific community has been searching for ways to reduce Cd toxicity. Many of the toxic effects of Cd are determined by its physical and chemical properties, and trace elements with chemical properties similar to those of Cd can interact with Cd. Such interactions can occur at different stages of trace element absorption, distribution, and excretion, affecting their biological function (17). The physical and chemical similarity between Cd and Zn suggests the existence of competitive antagonism. Zn plays key roles in gene expression and cell division and growth *via* various pathways and is essential for the proper functioning of many enzymes (111). Zn supplementation can reduce Cd damage to cell morphology and improve the activity of immune cells, such as macrophages and NK cells (76, 112). Jemai et al. showed that Cd exposure significantly reduced superoxide dismutase (SOD), and catalase (CAT) activities, triggering oxidative stress reactions, in rats. However, Zn supplementation significantly reduced the concentration of lipid peroxides and inhibited the increase in oxidative stress levels induced by Cd (17). Wang et al. have shown that hydrogen sulfide (H_2_S) antagonizes Cd^2+^ by regulating the antioxidant system, alleviating cell membrane damage, and maintaining intracellular homeostasis (113). In addition, H_2_S can also reduce inflammatory cell responses by inhibiting NF-κB pathway activation (114).

Similarly, selenium (Se) supplementation can promote the proliferation of immune cells, such as T-cells and NK cells (115, 116). Se has antioxidant properties and regulates immunity by functioning as a trace element (117, 118). Ge et al. have shown that Se nanoparticles weakened Cd-induced inflammatory responses through the NF-κB pathway (119). Se antagonizes Cd-induced toxicity *via* ROS-dependent oxidative stress and protects lymphocytes from Cd-induced apoptosis by inhibiting the PI3K/Akt pathway (120). Se antagonized ERS induced by Cd and effectively protected chicken neutrophils from changes caused by Cd (41). Metal elements and their organic compounds have dynamic regulation in cells (111). Iron (Fe) regulates the immune response in immune cells such as macrophages (121, 122). Chemical elements used for intervention may act antagonistically with Cd, improving Cd-induced immune damage. Metal ions (e.g., sodium (Na), Fe, and aluminum (Al)) can be combined with biosorption materials to improve their adsorption performance, and such materials can be used to remove Cd (123, 124).

(2) Block the activation of Cd-induced signaling pathways

Some of the ways to antagonize Cd toxicity are inhibiting ROS generation, reducing oxidative stress levels, maintaining redox balance, and inhibiting abnormal immune signaling activation. Bioactive compounds such as vitamins A and C, phenolic compounds, and flavonoids have good antioxidant activities or can improve antioxidant enzyme activity and thus reduce oxidative stress levels and inhibit signaling activation (125). Hyperoside inhibits LPS-induced inflammatory responses in microglial cells *via* the NF-κB pathway. Wild simulated ginseng activates RAW264.7 mouse macrophages through TRL2/4-dependent activation of the MAPK, NF-κB, and PI3K/Akt pathways. And vitamin C enhances the antioxidant ability of chicken myocardium cells to relieve heat stress injury (126–128). In addition, Cd exposure affects the composition of lymphocyte subsets and induces apoptosis of CD4 and CD8 cells. Fawzi et al. showed that multivitamins induced significant increases in CD4, CD8, and CD3 cell counts (129). Thus, in addition to trace elements, intervention with natural active ingredients or vitamins may inhibit the immunotoxicity of Cd.

Abnormal cytokine secretion and gene expression can affect normal cellular immune signaling activation. Cd affects cytokine expression and secretion by altering the activity of immune cells. For example, it promotes the expression of the pro-inflammatory factors IL-1β, IL-6, and TNF-α in macrophages by increasing ROS production, while inhibiting that of the anti-inflammatory cytokine IL-10 (46, 74). Therefore, blocking NF-κB signaling, oxidative stress, and the expression of pro-inflammatory factors, and promoting anti-inflammatory factors may be useful to alleviate Cd immunotoxicity. Catechin hydrate reduces Cd-related genotoxicity and cytotoxicity by inhibiting related apoptotic gene expression (130). Resveratrol protects cells from oxidative stress damage by activating NF-κB signaling (131). Hyperoside significantly reduces the expression of IL-1β and TNF-α through NF-κB signaling, thereby suppressing the inflammatory response of cells (127). In addition to naturally active products, some essential trace elements, such as Zn and Se, and vitamins perform similar biological functions in the body (e.g., anti-inflammatory and antiviral activities) through the NF-κB pathway. Se inhibits NF-κB pathway activation and reduces oxidative stress levels and promotes the expression of the anti-inflammatory cytokine IL-2 (132–134). Vitamins C, D, and E all regulate NF-κB signaling and have biological activity (135–137). The toxic effects of Cd on the spleen and B lymphocytes can be effectively reduced by inhibiting autophagy NLRP3 activity and by suppressing lymphocyte pyroptosis (71, 105).

Based on a better understanding of the pathways of Cd-induced immunotoxicity, interventions using inhibitors (e.g., metal ions, vitamins, and natural active products) can be designed and developed to effectively reduce Cd toxicity. While studying the immunosuppressive and toxic effects of Cd in the body, it is necessary to find effective measures to disrupt the activation of the mechanisms of Cd toxicity. Accordingly, reducing Cd accumulation in human organs and cells, alleviating its toxic effects, and reducing the risk of Cd-associated disease will be important research objectives in the future (**Table 3**) (41, 46, 84, 85, 105, 120).

**Table 3:** The detoxification mechanism to inhibit Cd immunotoxicity.

Cd and cells	Toxic effects on cells	Detoxification mechanism	Reference
Macrophages	Cd promotes macrophage TNF-α, IL-6, IL-1, NO, and catalase activity	Glycine reduces Cd-induced alterations in the viability and activation of macrophages	(84)
	Cd accumulated in macrophages and produces toxicity	Metallothionein intervention can be combined with Cd to inhibit Cd-induced toxicity	(85)
	Cd inhibits the phagocytotic activity of chicken peritoneal macrophages	Antagonistic effect of N-acetyl-L-cysteine against Cd-induced Cytotoxicity	(46)
	Cd caused dose-dependent morphologic and ultrastructural alterations in macrophages	Zn against Cd cytotoxicity in macrophages	(112)
Neutrophiles	Cd caused apoptosis by endoplasmic reticulum stress	Se can be antagonists with cd, effectively protecting chicken neutrophils from changes caused by Cd.	(41)
Natural killer (NK) cells	Cd can reduce the activity of NK cells	Zn can significantly increase the activity of NK cells	(76)
Lymphocytes	Cd caused apoptosis of exodus lymphocytes	Sulforaphane therapy restores Cd-induced apoptosis by 17% to 20%	(138)
	Cd caused autophagy and promoted apoptosis of B lymphocytes	Inhibit autophagy	(71)
	Cd promoted lymphocytes pyroptosis	Inhibitory NLRP3 activity	(105)
	Cd promoted apoptosis and necrosis of carp lymphocytes by regulating the miR-216a-PI3K/AKT axis	Se antagonizes Cd toxicity through ROS-dependent oxidative stress and PI3K/AKT pathways.	(120)
	Cd has cytotoxicity and genotoxicity to human lymphocytes	Catechin hydrate can inhibit the anti-genotoxicity and anti-cytotoxicity of Cd by inhibiting the expression of related apoptotic genes	(130)

## Summary and Perspectives

Cd enters the body through inhalation and ingestion. It accumulates in different various organs, tissues, and cells, and acts on immune organs and immune cells. Thus far to date, most studies investigating the effects of Cd exposure on immunomodulation have been committed to exploring the effects of focused on Cd toxicity ion the different organs, while; the changes that induced by Cd in immune cells undergo following Cd exposure, as well as its toxicity mechanisms and corresponding potential detoxification mechanisms, are less extensively studied.

When in immune cells, Cd dose-dependently affects cell vitality and functions and can induce apoptosis. Cd exposure impacts innate immunity in terms of phagocytic capacity, proliferation, and status transformation of macrophages, reduction of the number of NK cells, and the increase of neutrophils, thus resulting in an inflammatory response. Cd exposure affects adaptive immunity mainly by resulting in apoptosis of T-cells and B-cells. In B-cells, Cd exposure affects surface antigen expression and selectively inhibits the synthesis of antibodies. Thus, Cd exposure can directly affect the growth of immune cells and their function.

As immune cells grow, they become polarized, producing different cell phenotypes. Lymphocyte subset composition is closely related to the immune capacity of the immune system. Different lymphocyte subsets exhibit different sensitivities to Cd exposure. T subsets respond to the apoptogenic effects of Cd in the order CD8^+^ > CD4^−^CD8^−^ > CD4^+^CD8^+^ > CD4^+^ (36). The CD4^+^/CD8^+^ ratio changes proportionally to Cd concentration and exposure time. In addition, Cd exposure leads to a significant increase in the Th17 lymphocyte subset and significant decreases in the Th1 and Th2 lymphocyte subsets (11). Thus, lymphocyte subsets are affected by Cd in concentration- and exposure time-dependent manners.

Immune cells secrete certain cytokines upon internal or external stimulus. Cd can affect the secretion and expression of cytokines. For example, in M1 macrophages, Cd reduces IL-1β, IL-6, and TNF-α expression; in M2 macrophages, it reduces IL-6 and IL-10 expression; and in lymphocytes, it reduces IFN-γ, IL-2, and IL-4 production. In addition, Cd exposure is often closely related to inflammation. For example, Cd exposure can exert an inflammatory effect by inducing ROS production, reducing antioxidant enzyme activity, and activating oxidative stress and ERS pathways. Through the induction of abnormal cytokine secretion and inflammatory reactions, Cd exposure can also affect normal immune signaling pathways of cells, e.g., by activating the MAPK pathway and NF-κB-dependent gene expression. This affects the proliferation and phenotypic transformation of macrophages, which in turn leads to macrophage immune dysfunction. Activation of the PI3K/Akt pathway promotes redox imbalance and in turn triggers inflammation and lymphocyte apoptosis. Cd has similar chemical properties as Ca and thus can enter cells through Ca^2+^ channels to increase intracellular Ca concentrations. Cd-induced Ca^2+^ regulates the phosphorylation/dephosphorylation of c-Jun NH2-terminal kinase and p38 MAPK and modulates macrophage immune activity (139). Cd can interfere with the CREB1 pathway, affecting the distribution and stability of lymphocyte populations. Thus, Cd can affect cytokine secretion by affecting the growth of immune cells and subsequently activate abnormal immune signaling pathways to produce immunotoxic effects and damage the immune function of the body.

Few studies have focused on the toxicity and mechanisms of Cd in immune cells. Thus, there is an urgent need to unravel the toxic effects and mechanisms of Cd in immune cells and to develop effective immunotherapies to alleviate the toxic effects of Cd. Based on present data on the signaling and inflammatory pathways induced by Cd immunotoxicity, regulating cellular immune activities and normal cytokine secretion and reducing the activation of certain signaling pathways may effectively inhibit Cd immunotoxicity. In conclusion, Cd can inhibit innate and adaptive immunity, suppress immune system functions, and lead to the onset of various chronic diseases.

Therefore, for both occupational or non-occupational exposure to Cd, it is important to resume research to improve public health immunization and to reduce the toxic effects of Cd on the immune system. A continued search for active substances in natural products that inhibit Cd toxicity will facilitate the discovery of compounds that prevent Cd accumulation as well as Cd-related immune diseases.

## Author Contributions

ZW and YS drafted the manuscript. WY, QB, and HW drafted and revised the manuscript. All authors contributed to the article and approved the submitted version.

## Funding

This study was supported by grants from the National Nature Science Foundation (81630086, 82030099 and 81973078), National Key R&D Program of China (2018YFC2000700), Shanghai Municipal Human Resources and Social Security Bureau (2018060), Shanghai Public Health System Construction Three-Year Action Plan (GWV-10.1-XK15), the Major Science and Technology Innovation Program of Shanghai Municipal Education Commission (2019-01-07-00-01-E00059), the International Cooperation Project of Guangzhou Development Zone (2017GH11), and Innovative Research Team of High-Level Local Universities in Shanghai.

## Conflict of Interest

The authors declare that the research was conducted in the absence of any commercial or financial relationships that could be construed as a potential conflict of interest.

## Abbreviations

Al, Aluminum; Akt, Protein kinase B; B-cells, B lymphocytes; Ca, Calcium; CAT, Catalase; Cd, Cadmium; CYPs, Cytochrome P450s; CREB1, cAMP responsive element binding protein 1; DCs, Dendritic cells; ERS, Endoplasmic reticulum stress; FcγRIIB, Fc-gamma receptor II; Fe, Iron; GSH, Glutathione; HIF-1α, Hypoxia-inducible factor 1α; IL, Interleukin; IARC, International Agency for Research on Cancer; IG, Immunoglobulin; IL-12p70, Interleukin-12 by LPS-activated; IFN, Interferon; IP_3,_ Inositol 1,4,5-trisphosphate; Keap1, Kelch-like ECH-associated protein 1; LPS, Lipopolysaccharide; M1 macrophages, Classically activated macrophages; M2 macrophages, Alternatively activated macrophages; MAPK, Mitogen-activated protein kinase; MHC, Major histocompatibility complex; Na, Sodium; NLRP3, Nod-like receptor protein 3; NF-κB, Nuclear factor kappa B; NK cells, Natural killer cells; Nrf2, Nuclear factor erythroid 2-related factor 2; PFC, Plaque-forming cell; PI3K, Phosphoinositide 3-kinase; QHS, quantitative hemolysis; ROS, Reactive oxygen species; Se, Selenium; SOD, Superoxide dismutase; H_2_S, Hydrogen sulfide; TNF, Tumor necrosis factor; Th 1, T helper; T-cells, T lymphocytes; VDR, Vitamin D receptor; Zn, Zinc.
